# Properties and Microstructure of Na_2_CO_3_-Activated Binders Modified with Ca(OH)_2_ and Mg(OH)_2_

**DOI:** 10.3390/ma15051687

**Published:** 2022-02-24

**Authors:** Lilan Xie, Kaiwei Liu

**Affiliations:** 1 School of Materials and Architectural Engineering, Guizhou Normal University, Guiyang 550001, China; 2 Anhui Province Engineering Laboratory of Advanced Building Materials, Anhui Jianzhu University, Hefei 230601, China

**Keywords:** alkali-activated slag, one-part geopolymer, sodium carbonate, auxiliary activator, shrinkage

## Abstract

Delayed strength development and long setting times are the main disadvantageous properties of Na_2_CO_3_-activated slag cements. In this work, combined auxiliary activators of Ca(OH)_2_ and Mg(OH)_2_ were incorporated in one-part Na_2_CO_3_-activated slag binders to accelerate the kinetics of alkali activation. The properties and microstructure evolution were investigated to clarify the reaction mechanism. The results showed that the additions of auxiliary activators promoted the hardening of the pastes within 2 h. The 28 days compressive strengths were in the range of 39.5–45.5 MPa, rendering the binders practical cementitious materials in general construction applications. Ca(OH)_2_ was more effective than Mg(OH)_2_ in accelerating the kinetics of alkali activation. The dissolution of Ca(OH)_2_ released more OH^−^ and Ca^2+^ ions in the aqueous phase to increase alkalinity in the aqueous phase and promote the formation of the main binding gel phase of calcium-aluminosilicate hydrate (C-A-S-H). An increase in the Ca(OH)_2_/Mg(OH)_2_ ratios increased autogenous shrinkage and decreased drying shrinkage of the binders. The formation of a compact pore structure restricted the water evaporation from the binders during the drying procedure.

## 1. Introduction

Alkali-activated slag (AAS) cements are of growing interest as a clinker-free alternative to ordinary Portland cements (OPC), and the application has the potential to lower the CO_2_ footprint in the construction industry [1]. AAS binders gain their strengths via the reaction between ground granulated blast furnace slag (GBFS) and highly alkaline activators, which form the main binding gel phase of calcium-aluminosilicate hydrate (C-A-S-H) [2]. The properly designed formulations usually exhibit advantageous properties of rapid compressive strength development, high resistance to chemical attacks, and favorable thermal stability [3,4,5]. These properties enable the AAS cements to be used in many specific applications of rapid repair and radioactive waste immobilization, and high-temperature applications [6,7,8].

The nature and concentration of activators significantly impact the performance of AAS binders [9,10]. The concentrated aqueous alkali (i.e., Na and K) hydroxide or silicate solutions are widely used as the activators for preparing AAS binders, facilitating the dissolution of the glassy phases in slag, polymerization of the dissolved ionic species, and precipitation of the C-A-S-H gel phase [11]. However, these strong bases of activators are still carbon-intensive and expansive, contributing to the majority of energy consumption and carbon emission in the production of AAS cements [12,13]. Furthermore, these viscous and corrosive alkali solutions are not user-friendly [14,15]. This drives the development of one-part AAS cements [16,17]. The dry mixtures of cements are prepared by premixing the solid alkali sources with slag, and can be used similarly to OPC via the “just add water” process [16]. Therefore, there is an urgent need to search for more user- and environment-friendly solid activators [18].

Sodium carbonate (Na_2_CO_3_) is a cost-effective alternative to sodium hydroxide or silicate [19,20,21]. Na_2_CO_3_-activated slag binders are reported to achieve considerable compressive strength (i.e., 30–40 MPa) after 28 days of ambient curing [19,20]. However, with the excessive CO_3_^2−^ ions released from Na_2_CO_3_, the moderate alkalinity conditions developed in the pastes usually result in much longer setting times, up to around 4 days in some formulations, than the pastes activated with the strong bases of activators [19]. The Ca^2+^ dissolved from slag initially react with the CO_3_^2−^ to precipitate as CaCO_3_, and the formation of the C-A-S-H gel phase occurs after the CO_3_^2−^ ions are exhausted, accounting for the prolonged hardening process [18,19,22]. For the purpose of serving the one-part Na_2_CO_3_-activated slag binders as practical cementing materials in general applications, recent studies are extensively focused on the use of auxiliary activators to accelerate the kinetics of alkali activation [23,24,25,26,27,28]. Kovtun et al. [29] enhanced the 1 day compressive strength of Na_2_CO_3_-activated slag concretes cured at ambient temperature up to 25 MPa via the additions of slaked lime (Ca(OH)_2_) and silica fume. Akturk et al. [30] observed that only the 3% Ca(OH)_2_ addition promoted the hardening of the pastes within 6.5 h. Wang et al. [31] revealed that the CaO addition was beneficial for the formation of C-A-S-H. Gao et al. [32] suggested that the initial reaction between Ca(OH)_2_ and Na_2_CO_3_ was effective in removing the CO_3_^2−^ ions in the aqueous phase to enhance the alkalinity. Besides CaO and Ca(OH)_2_, some Mg-rich auxiliary activators have also been reported to be effective in expediting the reaction kinetics. Ke et al. [24] observed that the calcined layered double hydroxide (Mg_0.7_Al_0.3_O_1.15_) addition could work as the CO_3_^2−^ binding agent in the Na_2_CO_3_-activated slag binder via the formation of the hydrotalcite-like phase (Mg_4_Al_2_(OH)_12_CO_3_·3H_2_O). Yang et al. [33] prepared one-part Na_2_CO_3_-activated slag cements using calcined dolomite as the reaction kinetics controlling additive, which provided considerable amounts of reactive MgO and CaO in the binders to promote the formation of the hydrotalcite-like phase and the C-A-S-H gel phase.

Although the influences of auxiliary activators on the performance of Na_2_CO_3_-activated slag cements (e.g., setting times and compressive strengths) were initially investigated, many questions in the accelerated reaction mechanism have remained unsolved. The consumption of CO_3_^2−^ ions in the aqueous phase is a complex process, particularly when studying a specific system with the combined additions of Ca-rich and Mg-rich activators [18,26,33]. There are few studies evaluating the effectiveness of Ca-rich and Mg-rich auxiliary activators, so further investigation is required for optimizing the formulations. Additionally, despite the satisfactory mechanical performance and durability, the AAS binders commonly exhibit large autogenous and drying shrinkage. It is important to measure the volume stability of Na_2_CO_3_-activated slag cements before widespread field application. To cope with these challenges, we investigated the compressive strength and autogenous and drying shrinkage of one-part Na_2_CO_3_-activated slag cements modified with both Ca(OH)_2_ and Mg(OH)_2_. The phase evolution was determined using X-ray diffraction (XRD), Fourier-transform infrared spectroscopy (FTIR), and thermogravimetric analysis (TGA), and the structure evolution was evaluated using the nitrogen adsorption technique and scanning electron microscopy (SEM).

## 2. Experimental Methods

**Figure 1:**
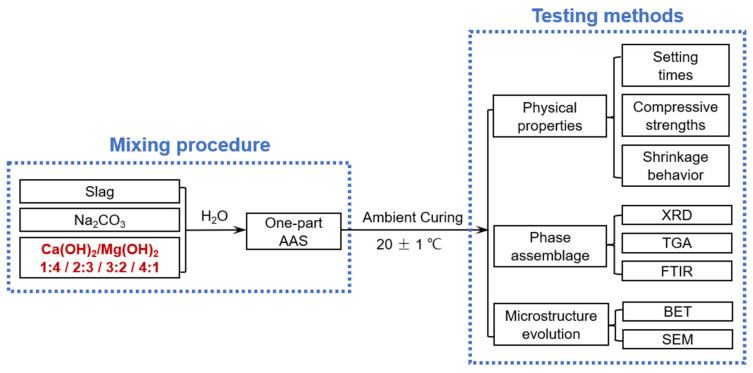
Schematic of the experimental procedure included in this study.

Figure 1 shows the schematic of the experimental procedure.

### 2.1. Mix Proportions

**Figure 2:**
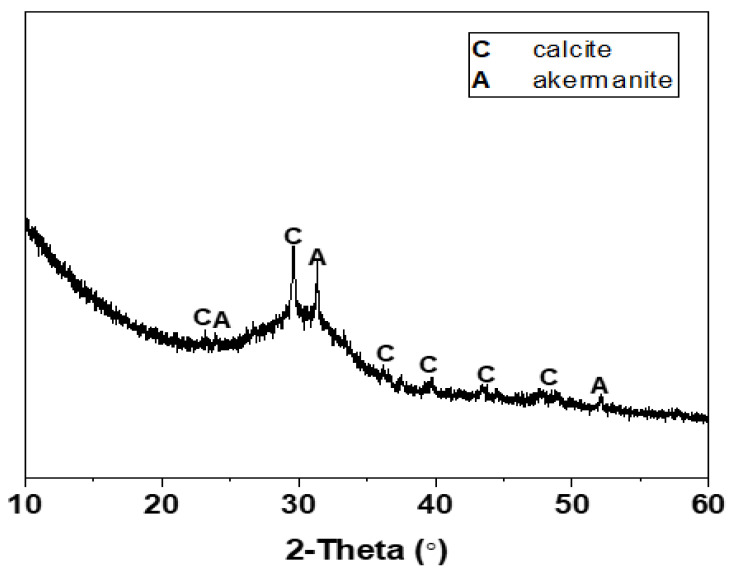
XRD pattern of GBFS.

The mix proportions of one-part Na_2_CO_3_-activated slag cements modified with the auxiliary activators of Ca(OH)_2_ and Mg(OH)_2_ are tabulated in Table 1. The chemical compositions of Grade S105 ground granulated blast furnace slag (GBFS) are listed in Table 2. The average particle size (d_50_) of slag analyzed using the laser diffraction was 12.46 μm. The XRD pattern of slag is shown in Figure 2. The broad diffraction hump distributed in the range of 25–35° 2θ indicated its amorphous nature, along with the main crystalline phases of calcite (CaCO_3_, PDF No. 72–1652) and akermanite (Ca_2_MgSi_2_O_7_, PDF No. 35-0592). The solid activators used in this work included the reagent-grade Na_2_CO_3_, Ca(OH)_2_ and Mg(OH)_2_. The dosage of Na_2_CO_3_ was fixed as 10 wt% of slag [19,25,33]. The blended Ca(OH)_2_ and Mg(OH)_2_ were used as the auxiliary activators with the purpose of expediting the kinetics of alkali activation [32]. The total amount of the blended Ca(OH)_2_ and Mg(OH)_2_ was fixed as 5 wt% of slag, which was chosen according to other studies of Na_2_CO_3_-activated slag binders to achieve reliable early-age strength development during ambient curing [23,24,33]. Their dosages were set as the mixture variables to investigate the potential effects of Ca(OH)_2_/Mg(OH)_2_ mass ratios (1:4, 2:3, 3:2, and 4:1) on the properties of binders. A constant water to binder (slag) ratio w/b of 0.50 was used to obtain the workable paste.

The slag and solid activators (Na_2_CO_3_, Ca(OH)_2_ and Mg(OH)_2_) were first dry-mixed for 4 min, and then mixed with water in a cement paste mixer for 4 min to obtain the fresh pastes. The Vicat method (ASTM C191-08 [34]) was used to measure the initial and final setting times of the pastes. The pastes were cast in cubic (30 mm × 30 mm × 30 mm) and prismatic (20 mm × 20 mm × 80 mm) polypropylene molds for testing the compressive strengths and length changes of the binders, respectively. The upper surfaces of molds were covered with glass plates, and then cured in a chamber (20 ± 1 °C).

### 2.2. Testing Methods

#### 2.2.1. Compressive Strengths and Shrinkage Behavior

After 24 h of curing, the hardened specimens were demolded. A length comparator was used to measure the volume change along the longitudinal axis. The initial lengths of the prismatic specimens were measured. Parts of the prismatic specimens were wrapped with polyethylene film to prevent moisture egress, and further cured in the chamber to measure the autogenous shrinkage. The other parts of the prismatic specimens were placed in the drying curing chamber (relative humidity = 60 ± 1% and 20 ± 1 °C) to measure the drying shrinkage. The linear shrinkage ratio (%) was calculated based on Equation (1), where *L_I_* (mm) is the initial length after demolding, *L_F_* (mm) is the measured length at the specific curing ages, and 75 (mm) is the effective length between the two head nails.

(1) $$Shrinkage=\frac{L_{I}-L_{F}}{75}\times 100\%$$

Mass loss associated with the water evaporation occurred in the prismatic specimens stored in the drying curing chamber was also tested and calculated based on Equation (2), where *M_I_* (mm) is the initial weight after demolding and *M_F_* (mm) is the measured weight at the specific curing ages. Four replicates were measured for obtaining the average value and standard deviation for the shrinkage and mass loss.

(2) $$Mass\;Loss=\frac{M_{I}-M_{F}}{M_{I}}\times 100\%$$

Additionally, the cubic specimens were still cured at the moisture chamber (relative humidity ≥ 95 ± 2% and 20 ± 1 °C) until the specific curing ages (1, 3, 7, and 28 days) for measuring the compressive strengths. Three replicates were tested for each mixture.

It should be noted that the binders without the addition of auxiliary activators (i.e., C0) exhibited a delayed strengths development. The paste was not strong enough to be handled or demolded even after 24 h of initial curing at ambient temperature. Therefore, only the later-age (i.e., 3, 7, and 28 days) compressive strengths were tested and are discussed in the following sections.

#### 2.2.2. Phase Assemblage and Microstructure

Characterization of phase assemblage of the binders was performed using XRD, TGA, and FTIR. After 28 days of curing, the prismatic specimens wrapped with polyethylene film were crushed into small pieces, immersed in isopropanol for 24 h to terminate hydration, dried at 60 °C for 3 h, and finely ground to obtain the powder samples. The mineralogical compositions were analyzed using the X-ray diffractometry (XRD, X’Pert^3^ Powder, PANalytical, Malvern, UK) method with CuKa radiation of 10–60° 2θ. The thermal decompositions were analyzed using the thermogravimetric analysis (TGA, STA 499C, NETZSCH, Selb, Germany) under the nitrogen atmosphere with a heating rate of 10 °C/min from 40 to 1000 °C. Fourier-transform infrared spectroscopy (FTIR, NEXUS-670 NICOLET, Leicester, UK) was collected in the spectral range from 400 to 2000 cm^−1^ using the potassium bromide (KBr) pellet method.

Characterization of microstructure of the binders was performed using the nitrogen adsorption technique and SEM. The pore structure of the granular sample with 1 ± 0.5 mm diameter was analyzed using the nitrogen adsorption technique (TriStar II 3020 instrument, Micromeritics, Norcross, GA, USA) with the Brunauer–Emmett–Teller (BET) method. The microstructure of the binders was analyzed using a field emission scanning electron microscope (SEM, Nova NanoSEM 650, FEI, Hillsboro, OR, USA). The fractured samples (around 5 mm diameter) were impregnated in epoxy, polished using the SiC paper and diamond powder sprays, and then coated with gold for the backscattered electron (BSE) imaging characterization. Accelerating voltage was set as 15 kV.

## 3. Results and Discussion

### 3.1. Setting Times and Compressive Strengths

**Figure 3:**
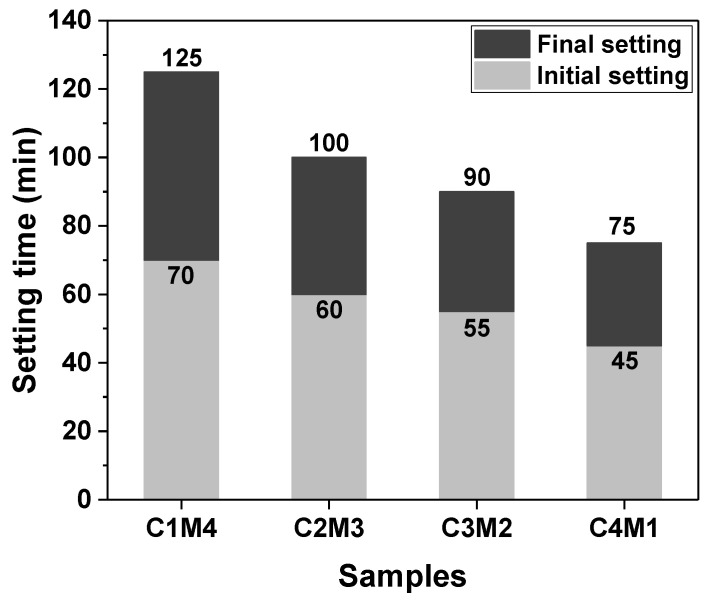
Initial and final setting times of the Na_2_CO_3_-activated slag pastes modified with Ca(OH)_2_ and Mg(OH)_2_.

The setting times of Na_2_CO_3_-activated slag binders modified with Ca(OH)_2_ and Mg(OH)_2_ are shown in Figure 3. Since the C0 paste exhibited delayed hardening even after 24 h of initial curing at ambient temperature, its setting times are not discussed in this section. The additions of auxiliary activators (Ca(OH)_2_ and Mg(OH)_2_) promoted the hardening of the pastes, and consequently, the final setting times were shortened to around 2 h. The initial and final setting times decreased linearly as the Ca(OH)_2_/Mg(OH)_2_ ratios increased, meaning that the Ca(OH)_2_ addition was more efficient in accelerating the reaction kinetics than the Mg(OH)_2_ addition. This is probably attributed to Ca(OH)_2_ having a higher solubility than Mg(OH)_2_ [35]. The higher concentration of OH^−^ ions released from Ca(OH)_2_ accelerates the dissolution of slag.

**Figure 4:**
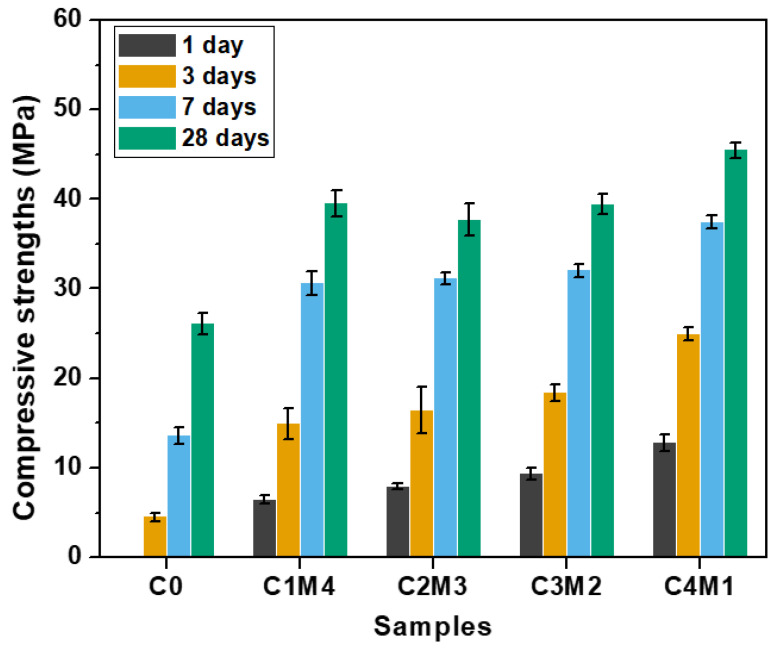
Compressive strengths of the Na_2_CO_3_-activated slag binders modified with Ca(OH)_2_ and Mg(OH)_2_.

The compressive strengths of Na_2_CO_3_-activated slag binders at the specific curing ages of 1, 3, 7, and 28 days are shown in Figure 4. The C0 binder activated solely with Na_2_CO_3_ exhibited delayed strength development. The 1 day compressive strength was negligible, and the 3 days compressive strength only reached 4.5 MPa. In comparison, the 1 day compressive strengths of the binders modified with the auxiliary activators were in the range of 6.5–12.8 MPa. This confirmed the efficiency towards accelerating the reaction kinetics. The 7 days compressive strengths were in the range of 30.6–37.5 MPa, which were close to the 28 days compressive strengths of 39.5–45.5 MPa. This indicates that these formulations exhibited rapid early-age strength development under the ambient curing. The compressive strengths at the specific curing ages increased almost linearly with the increasing Ca(OH)_2_/Mg(OH)_2_ ratios. Such an observation is consistent with the current state of knowledge that the addition of a Ca-rich auxiliary activator is beneficial for the formation of the main binding gel phase of C-A-S-H in the binders [31,32]. Additionally, the initial reaction between Na_2_CO_3_ and Ca(OH)_2_ results in the CaCO_3_ precipitation in the aqueous phase, and thus, the presence of additional surfaces probably improves the nucleation and growth of the C-A-S-H gel phase [36]. It should be noted that the 28 days compressive strength (26.1 MPa) of C0 was lower than the strengths of binders modified with auxiliary activators. The Ca(OH)_2_ and Mg(OH)_2_ additions also enhanced the degree of alkali activation at the later curing age.

### 3.2. Autogenous and Drying Shrinkage

**Figure 5:**
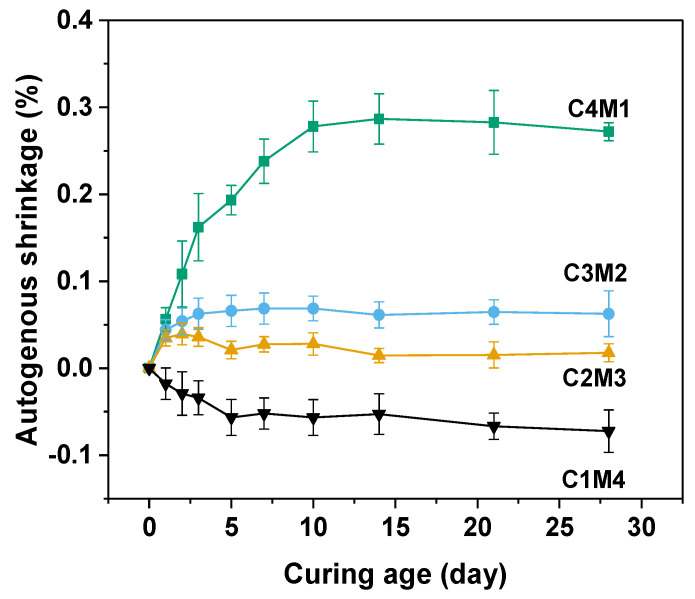
Autogenous shrinkage of the Na_2_CO_3_−activated slag binders modified with Ca(OH)_2_ and Mg(OH)_2_.

Autogenous shrinkage of the cementitious material is driven by the chemical shrinkage and self-desiccation [37,38]. In this work, the initial lengths of prismatic specimens were measured after 24 h of hardening. The self-desiccation means the pore water consumption occurred during the continuous alkali activation. Free water present in the aqueous phase is consumed and chemically bound in the newly formed main binding gel phase of C-A-S-H [38]. Due to the formation of water–air menisci and a capillary pore network, tensile stress and capillary pressure developed in the hardened binder, resulting in the autogenous shrinkage behavior [39]. Figure 5 shows the autogenous shrinkage curves of Na_2_CO_3_-activated slag binders. The 28 days autogenous shrinkage values consistently increased from −0.0723% to 0.2720% with the increasing Ca(OH)_2_/Mg(OH)_2_ ratios, indicating that the autogenous shrinkage was more pronounced in the binders with higher dosages of Ca(OH)_2_. This observation was consistent with the previous observation that the self-desiccation was mostly determined by the degree of alkali activation [39]. It should be noted that the C1M4 binder exhibited slight expansion after 28 days of ambient curing, probably because the excessive addition of Mg(OH)_2_ acted as the expansive agent in the binder to mitigate the volume shrinkage [40].

**Figure 6:**
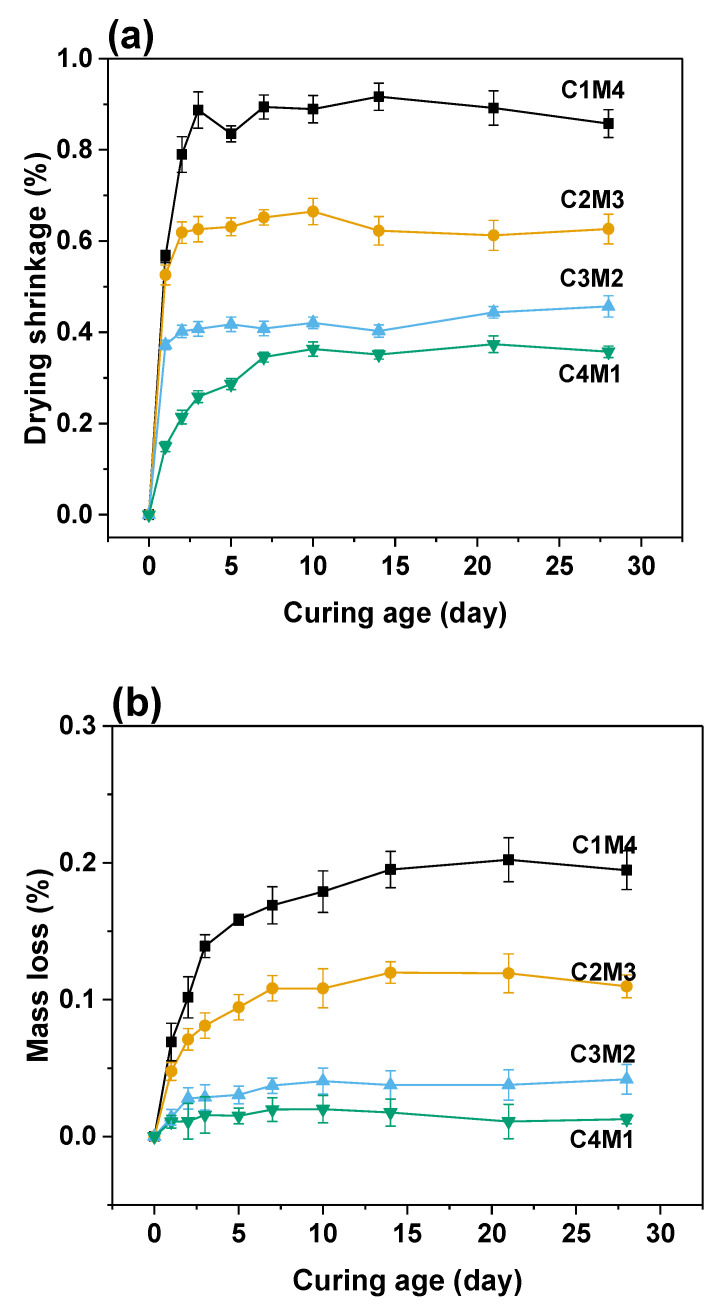
(**a**) Drying shrinkage and (**b**) mass loss of the Na_2_CO_3_-activated slag binders exposed to 60% relative humidity environment.

The drying shrinkage originates from the moisture loss in the hardened binder suffered the external evaporation. The water–air menisci developed in the capillary pores result in the capillary stress, so the drying shrinkage is significantly influenced by the pore structure [41,42]. As shown in Figure 6a, the 28 days drying shrinkage values were in the range of 0.3572–0.8577%, nearly 3–4 times higher than the autogenous shrinkage. In contrast to the trend identified in the autogenous shrinkage, the drying shrinkage values consistently decreased with the increasing Ca(OH)_2_/Mg(OH)_2_ ratios. Such an observation agrees well with the mass loss variation shown in Figure 6b. The 28 days mass loss ratios consistently decreased from 0.1947% to 0.0127% with the increasing Ca(OH)_2_/Mg(OH)_2_ ratios. The strong interrelationship between the drying shrinkage and moisture loss indicates that more C-A-S-H gel phase is formed in the binders with higher dosages of Ca(OH)_2_ to compact the pore structure, and consequently, the water evaporation during the drying procedure is restricted.

### 3.3. Phase Assemblage

#### 3.3.1. XRD

**Figure 7:**
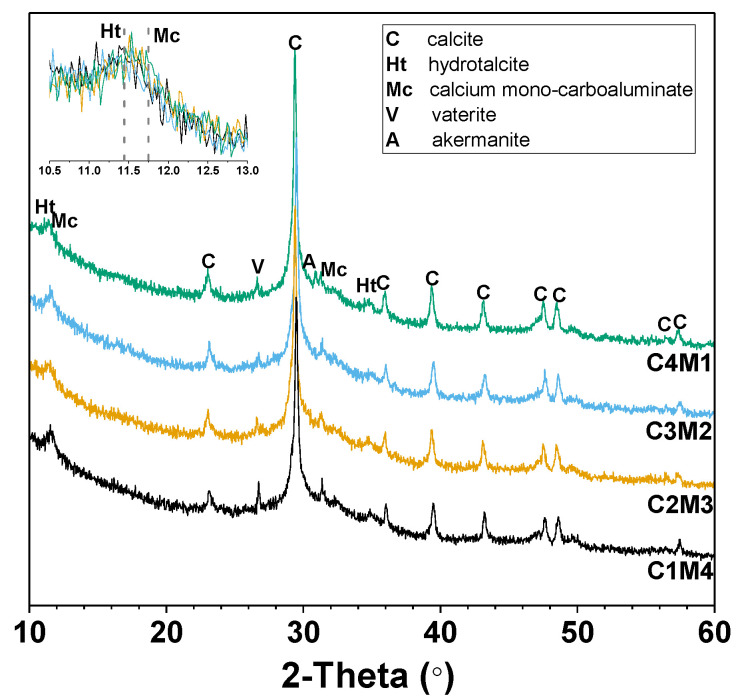
XRD patterns of the Na_2_CO_3_-activated slag binders modified with Ca(OH)_2_ and Mg(OH)_2_.

Figure 7 shows the XRD patterns of the 28 days cured Na_2_CO_3_-activated slag binders modified with Ca(OH)_2_ and Mg(OH)_2_. The amorphous hump distributed in the range of 25–35° 2θ is mainly assigned to the amorphous phase derived from the slag remnants. This also suggests the formation of the main binding gel phase of C-A-S-H as one of the main reaction products [43]. The intensities of the peaks assigned to calcite (CaCO_3_, PDF No. 72–1652) increase with the increasing Ca(OH)_2_/Mg(OH)_2_ ratios of the auxiliary activators, mainly attributed to the initial reaction between Na_2_CO_3_ and Ca(OH)_2_. Calcium carbonate also precipitates as different polymorphs of vaterite (CaCO_3_, PDF No. 72–0506) [24]. The additional crystalline phases identified in the binders included the hydrotalcite-like phase (Mg_6_Al_2_CO_3_(OH)_16_·4H_2_O, PDF No. 41-1428) and calcium mono-carboaluminate (Ca_4_Al_2_O_6_CO_3_·11H_2_O, PDF No. 41-0219), both of which are members of AFm phases, and their key Bragg reflections overlapped in the range of 11–12° 2θ [24,44]. Traces of akermanite (Ca_2_MgSi_2_O_7_, PDF No. 35-0592) are derived from the slag remnants [24]. The Mg(OH)_2_ addition has a dominant contribution to the formation of the hydrotalcite-like phase in the binders [25]. It is interesting to note that brucite (Mg(OH)_2_) is not identified in all the binders, even in the mixtures with relatively higher dosages of Mg(OH)_2_. This confirms its consumption during the formation of the hydrotalcite-like phase.

#### 3.3.2. TGA

**Figure 8:**
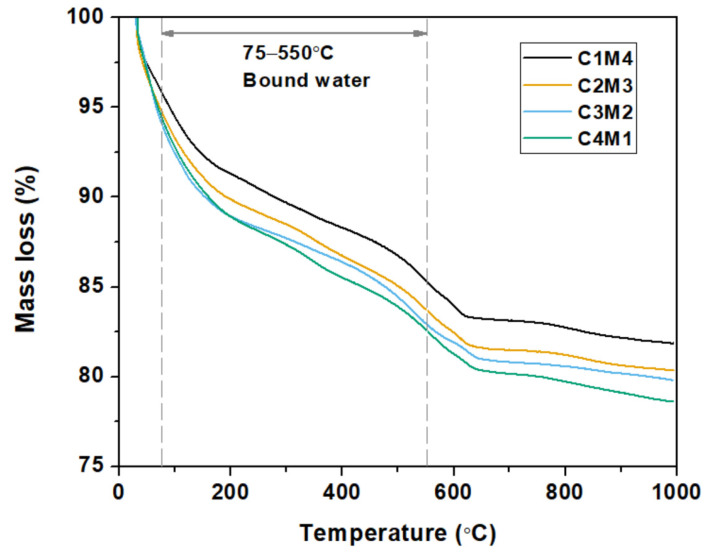
Thermogravimetric curves of the Na_2_CO_3_ –activated slag binders modified with Ca(OH)_2_ and Mg(OH)_2_.

Figure 8 shows the thermogravimetric curves of the 28 days cured Na_2_CO_3_-activated slag binders. For the purpose of investigating the influences of Ca(OH)_2_/Mg(OH)_2_ ratios on the degree of alkali activation, the bound water contents in the binders (mainly in the C-A-S-H gel phase) were calculated based on the TGA data [45]. As tabulated in Table 3, the bound water content consistently increases with the increasing Ca(OH)_2_/Mg(OH)_2_ ratios. This agrees well with our speculation that the increased Ca(OH)_2_ dosage enhances the degree of alkali activation. The Ca^2+^ ions dissolved from Ca(OH)_2_ promote the formation of C-A-S-H type gel. Meanwhile, the precipitation of calcite in the binder appears to improve the nucleation and growth of the C-A-S-H gel phase [46].

#### 3.3.3. FTIR

**Figure 9:**
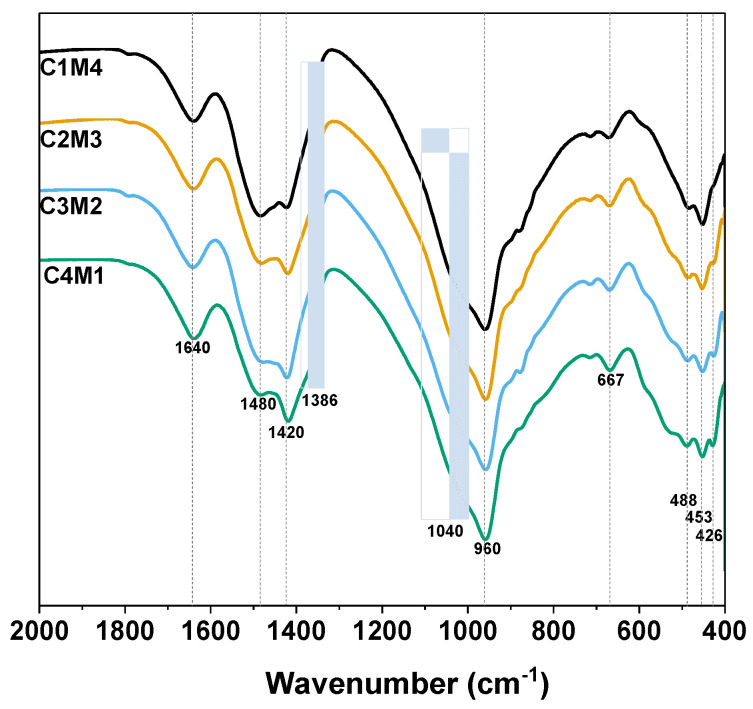
FTIR spectra of the Na_2_CO_3_ –activated slag binders modified with Ca(OH)_2_ and Mg(OH)_2_.

Figure 9 shows the FTIR spectra of the 28 days cured Na_2_CO_3_-activated slag binders. The broad bands centered at around 1640 cm^−1^ are assigned to the H-O-H bending vibrations of bound and interlayer water in the C-A-S-H gel phase, as well as the crystal water in the hydrotalcite-like phase and AFm phases [47]. The ν_3_ stretching vibration bands of CO_3_^2−^ at approximately 1480 cm^−1^ and 1420 cm^−1^ are associated with the typical carbonate products of calcite and vaterite formed in the binder, and the shoulder that appeared at around 1386 cm^−1^ is assigned to the CO_3_^2−^ in the calcium mono-carboaluminate [48]. The significant vibration located at around 960 cm^−1^ is assigned to the T-O asymmetric stretching vibrations (T = Si or Al) (typical Q^2^ units of silica tetrahedron) in the C-A-S-H type gel [49]. The shoulder at around 1040 cm^−1^ seems to be associated with the Q^3^ sites of highly polymerized silicate species [50]. The bands at 667 cm^−1^, 488 cm^−1^, 453 cm^−1^ and 426 cm^−1^ are also assigned to the Si-O-Si bending vibration [47].

### 3.4. Microstructure Evolution

#### 3.4.1. Pore Structure

**Figure 10:**
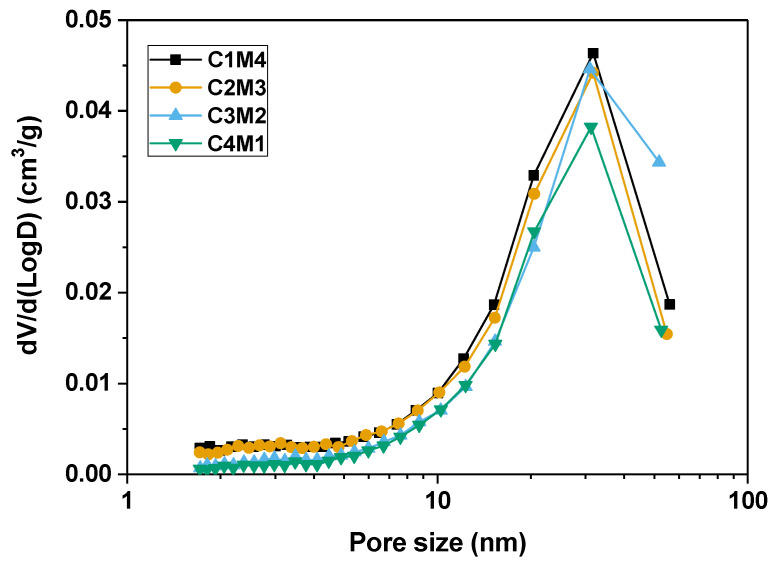
Pore size distribution curves of the Na_2_CO_3_ –activated slag binders modified with Ca(OH)_2_ and Mg(OH)_2_.

Figure 10 shows the pore size distribution curves of the 28 days cured binders determined by the nitrogen adsorption technique using the Brunauer–Emmett–Teller (BET) method, and the pore structure parameters are tabulated in Table 3. Although no clear variation trend is identified in the average pore diameter, the cumulative pore volume decreases linearly as the Ca(OH)_2_/Mg(OH)_2_ ratio increases. This supports our speculation that the Ca(OH)_2_ addition promotes the formation of the C-A-S-H gel phase to fill the pore structure. Additionally, the delayed carbonation of Mg(OH)_2_ and the formation of hydrated magnesium carbonates probably have detrimental effects on the pore structure compactness of the binders [51]. Previous research indicates that the more compact pore structure restricts the water movement to the exposed surface, and meanwhile breaks the hydraulic connection between the evaporation zone and the internal saturated space, resulting in less water evaporation [52]. Fewer menisci developed in the capillary pores reduce the capillary pressure and drying shrinkage in the binders with relatively higher Ca(OH)_2_/Mg(OH)_2_ ratios.

#### 3.4.2. SEM

**Figure 11:**
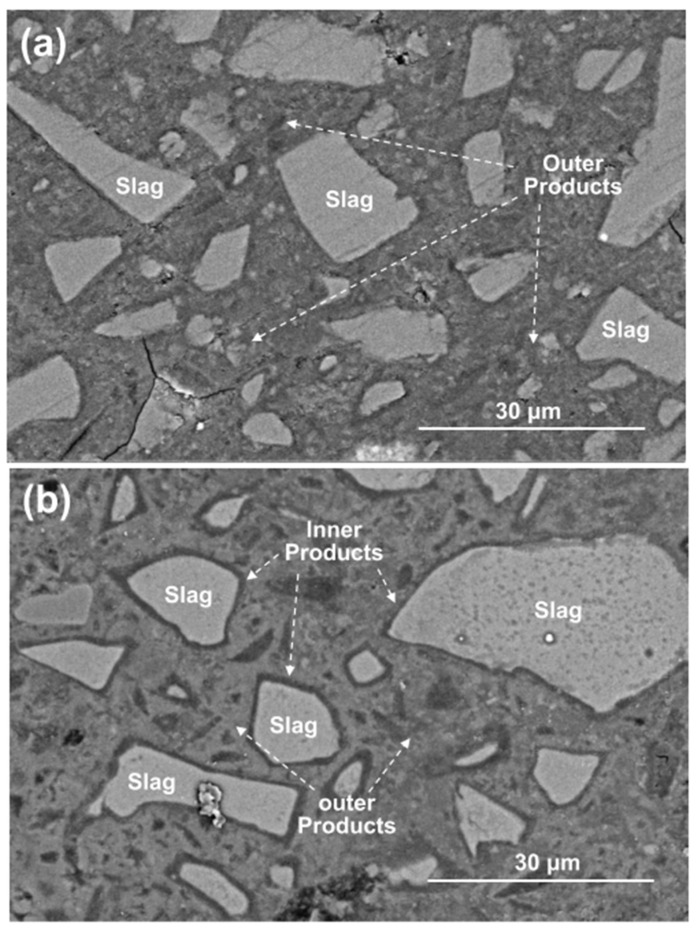
BSE images of the 28 days cured (**a**) C1M4 and (**b**) C4M1 samples.

Figure 11 shows the BSE images of polished cross-sections in the 28 days cured C1M4 and C4M1 samples. Like the typical morphology of the AAS binders, the light gray slag remnants are distributed in the dense and cohesive binding matrix, commonly recognized as the outer products consisting mainly of the C-A-S-H type gel [53,54]. It should be noted that the dark gray reaction product rims covered on the surface of slag remnants (inner products) are only identified in the C4M1 sample. The inner product layer is commonly developed in the position of the original slag grains, indicating a higher dissolution degree of the slag particles [53]. In comparison, the C1M4 sample has a less compact microstructure with some microcracks propagation in it.

Based on the phase assemblage and microstructure evolution results discussed above, the acceleration mechanism in the Na_2_CO_3_-activated slag binders modified with the combined auxiliary activators (Ca(OH)_2_ and Mg(OH)_2_) is proposed here. The Mg^2+^ ions released from Mg(OH)_2_ react with the Al^3+^ ions dissolved from slag to form the hydrotalcite-like phase, which has the Mg-Al double layered structure and works as the carbonate binding agent in the binder to increase the alkalinity [25]. When compared with Mg(OH)_2_, Ca(OH)_2_ is more effective in accelerating the reaction kinetics. Due to the higher solubility of Ca(OH)_2_, more OH^-^ ions are released in the paste to yield a significant increase in the pH, resulting in a further dissolution of slag. More Ca^2+^ ions released from Ca(OH)_2_ are able to promote the formation of the main binding C-A-S-H gel in the binder. The formation of a compact pore structure not only enhances the compressive strengths, but also mitigates the drying shrinkage of the binders.

## 4. Conclusions

In this work, the combined auxiliary activators of Ca(OH)_2_ and Mg(OH)_2_ were incorporated in the Na_2_CO_3_-activated slag binders to promote hardening and accelerate the early-age strength development at the ambient temperature. The setting times, compressive strengths, and autogenous and drying shrinkage were evaluated, and the phase assemblage and microstructure evolution were characterized via a variety of methods. The main conclusions are listed as follows.

The Ca(OH)_2_ addition was more effective than Mg(OH)_2_ in accelerating the reaction kinetics. The 28 days compressive strengths increased from 39.5 MPa to 45.5 MPa when the Ca(OH)_2_/Mg(OH)_2_ ratios were further increased. The hardening of the pastes was promoted within 2 h. These mechanical properties mean the Na_2_CO_3_-activated slag binders are sufficient to serve as practical cementitious materials in general construction applications. Besides the setting times and compressive strengths, the additions of auxiliary activators were also significant in determining the shrinkage behavior of binders. The autogenous shrinkage of binders increased, while the drying shrinkage decreased consistently with the increasing Ca(OH)_2_ dosages. A higher degree of alkali activation led to higher autogenous shrinkage. Meanwhile, the Ca(OH)_2_ addition promoted the formation of the C-A-S-H gel phase to compact the pore structure. This restricted the water evaporation during the drying procedure.

These findings may help us to understand the reaction mechanisms in the Na_2_CO_3_-activated slag binders incorporated with auxiliary activators and further optimize the formulations. This will be helpful in making such material into viable and large-scale engineering applications. Although the production of these auxiliary activators slightly enhances energy requirements and CO_2_ emissions, the one-part AAS formulations proposed here are more environmentally friendly and cost-effective than the binders prepared with strong bases of activators. Future work is required to clarify the long-term durability of such material, such as its resistance to carbonation and sulphate attack.

## Figures and Tables

**Table 1:** Mix proportions of one-part Na_2_CO_3_-activated binders.

Mixtures ^a^	GBFS (g)	Na_2_CO_3_ (g)	Ca(OH)_2_ (g)	Mg(OH)_2_ (g)	Water (g)	w/b	Na:Al	Ca/Si	Mg/Si
C0	100	10	-	-	50	0.5	0.54	1.18	0.26
C1M4	100	10	1	4	50	0.5	0.54	1.29	0.39
C2M3	100	10	2	3	50	0.5	0.54	1.32	0.36
C3M2	100	10	3	2	50	0.5	0.54	1.34	0.33
C4M1	100	10	4	1	50	0.5	0.54	1.37	0.29

^a^ The mixing codes of formulations were defined as the dosages of Ca(OH)_2_ and Mg(OH)_2_ by weight of slag, e.g., C1M4 for the binders activated with 10 wt% Na_2_CO_3_, 1 wt% Ca(OH)_2_, and 4 wt% Mg(OH)_2_, while C0 for the binders activated solely with 10 wt% Na_2_CO_3_.

**Table 2:** Chemical compositions (wt%) of GBFS.

Materials	SiO_2_	Al_2_O_3_	CaO	MgO	Fe_2_O_3_	SO_3_	Na_2_O	K_2_O	TiO_2_	LOI ^a^
GBFS	32.2	18.6	38.1	5.6	0.7	1.1	0.3	0.6	0.7	2.1

^a^ LOI is the loss on ignition at 1000 °C.

**Table 3:** Bound water and pore structure parameters of the Na_2_CO_3_-activated slag binders modified with Ca(OH)_2_ and Mg(OH)_2_.

Samples	Bound Water (%)	Pore Volume (×10^−3^ cm^3^/g)	Average Pore Diameter (nm)
C1M4	12.35	40.78	20.37
C2M3	13.22	36.63	19.79
C3M2	13.50	37.16	23.16
C4M1	14.41	33.53	22.33

## Data Availability

The data presented in this study are available on request from the corresponding author.
